# Differential Patterns of Cognitive Complaints in Mild Cognitive Impairment and Subjective Cognitive Decline in Individuals Born in Canada or Abroad

**DOI:** 10.1002/alz70857_098909

**Published:** 2025-12-24

**Authors:** Astrid Coleman, Renée K Biss, Ana Badal, Kristoffer Romero

**Affiliations:** ^1^ University of Windsor, Windsor, ON, Canada; ^2^ York University, North York, ON, Canada

## Abstract

**Background:**

Subjective cognitive complaints (SCCs) play an important role in diagnosing mild cognitive impairment (MCI), but their utility in differentiating MCI and normal aging remains unclear. Moreover, the impact of cultural factors on SCCs has largely been overlooked. Therefore, the goal of the current study was to explore whether certain SCCs more strongly predict MCI or intact cognitive functioning and whether these effects differ for individuals born in Canada compared to those born abroad.

**Method:**

The current retrospective study examined older adults (aged 60‐94) referred to a tertiary memory clinic in Toronto, Canada, who were given a comprehensive neuropsychological evaluation. Participants either met criteria for MCI (*n* = 74) or had SCCs but intact cognition, meeting research criteria for subjective cognitive decline (SCD; *n* = 60). Participants were also classified by birthplace (Canada: *n* = 66; abroad: *n* = 68). SCCs were assessed using the Abilities subscale of the Multifactorial Memory Questionnaire (MMQ), a 20‐item self‐report measure that examines self‐perception of memory abilities in daily life.

**Result:**

The overall frequency of SCCs (i.e., total MMQ score) did not differ significantly by clinical group (MCI or SCD) or birthplace (Canada or abroad). Logistic regression analyses based on individual MMQ items revealed several questions that predicted clinical group classification or birthplace. In general, SCCs regarding prospective memory were more frequent in patients born in Canada vs. those born abroad. Within groups, SCCs of memory loss for well‐learned semantic information differentiated SCD vs. MCI for individuals born in Canada. No SCCs differentiated SCD vs. MCI in those born abroad.

**Conclusion:**

Summary scores of SCCs are not strongly associated with objective cognitive functioning: However, certain SCCs were more frequent in patients born in Canada vs. abroad, and specific cognitive complaints significantly differentiated clinical groups as a function of birthplace (i.e., long‐term memory complaints predicting MCI in Canada‐born individuals only). These findings demonstrate the need to refine SCC measures to optimize clinical utility, and also underscore the importance of considering cultural factors (i.e., birthplace) and how they may impact clinical presentation when assessing for cognitive decline in older adults.

